# Supplementary material for: Trackable and scalable LC-MS metabolomics data processing using asari
Source: Nat Commun. 2023 Jul 11;14:4113. doi: 10.1038/s41467-023-39889-1 (PMC10336130; doi:10.1038/s41467-023-39889-1)
Supplement: Supplementary file 1 — Supplementary Information [file 41467_2023_39889_MOESM1_ESM.pdf]

Supplementary Information:

Trackable and scalable LC-MS metabolomics data processing using asari

Shuzhao Li*, Amnah Siddiqa, Maheshwor Thapa, Yuanye Chi and Shujian Zheng
Jackson Laboratory for Genomic Medicine, 10 Discovery Drive, Farmington, CT 06032, USA
*Corresponding author, E-mail: shuzhao.li@jax.org

Supplementary Methods

Software design of asari.

mSelectivity.

Metabolomics datasets.

LC-MS metabolomics experiments.

LC-MS metabolomics data processing.

Evaluation of feature detection.

Evaluation of computational performance.

Data structures in asari.

Step-by-step description of asari default processing workflow.

Supplementary Tables

Supplementary Table 1. Pairwise, unambiguously matched features between processing tools on the HZV029 dataset.

Supplementary Table 2. Pairwise, unambiguously matched features between processing tools on the Yeast2021 dataset.

Supplementary Figures

Supplementary Figure 1 Illustration of mass tracks in a single sample.

Supplementary Figure 2. Composite mass track enhances peak detection by aggregating signals from all samples.

Supplementary Figure 3. Manually verified features that are not reported by asari in the HZV029 dataset (corresponding to Figure 4a).

Supplementary Figure 4. Manually verified features that are not reported by MS-DIAL in the HZV029 dataset (corresponding to Figure 4a).

Supplementary Figure 5. Four manually verified features are not reported by asari in the Yeast2021 dataset (corresponding to Figure 4b).

Supplementary Figure 6. Correlation of 13C isotopologues and Na+ adducts to the known compounds in the Yeast2021 dataset (corresponding to Figure 4b).

Supplementary Figure 7. Features not reported by asari in the SZ22 E. coli dataset (corresponding to Figure 4c).

Supplementary Figure 8. Example chromatograms for illustrating issues in peak detection.

Supplementary Figure 9. Example of QC overview with many low-quality peaks.

Supplementary Methods

Software design of asari. Asari is written in Python 3, and can be used as a standalone command line tool or imported as a package. Its library dependency includes numerical computing via numpy and scipy, data wrangling via pandas, and visualization via panel and hvplot. Pymzml is used to parse mzML format. Data structures, annotation, search and chemical calculation make use of our supporting packages metDatamodel, mass2chem and jms-metabolite-services. Implementation of new and previous algorithms was coded from the ground up, where numerous details contributed to the computing speed, e.g., discrete mathematics is preferred over continuous curves, and intermediary indexing and caches are employed. Processed mass tracks are cached on disk to reduce memory footprint. Mass tracks are explicitly linked with features and peaks, and the information is exported as JSON in asari output. The quality metric mSelectivity is used internally. Other quality metrics, peak shape, SNR and cSelectivity, are part of exported feature tables. Grouping ions into empirical compounds is performed using khipu (Li and Zheng[1]). The annotation and search functions are generic to accommodate reference databases, and the default is HMDB[2].

mSelectivity. The probability of confusing two m/z values *i* and *j* is modelled as

$$P_{i,j} = e^{\frac{-\Delta m/z}{\xi}}$$

, where ξ is the preset mass resolution, e.g. 5 ppm. The mSelectivity of m/z value *i* among a list of *n* values is defined as

$$\prod_{1 \leq k \leq n,\ \ k \neq i}^{n} (1 - P_{i,k})$$

. In practice, we only need to calculate the mSelectivity based on the two neighbors of higher m/z values and two of lower m/z values to obtain an accurate approximation.

Metabolomics datasets

Four new datasets were generated in this study: HZV029 (human plasma samples), MT02 (human plasma samples), SZ22 (E. coli samples with 13C isotope labeling), and BM21(serial mixture of human plasma and vegetable juice). In HZV029, 184 samples were used for analysis here (additional samples of different source included in data repository). Four public datasets are used here: Yeast2021 (Chen *et al.*[3]), SLAW (E. coli, large Orbitrap QE data as described in

Delabriere *et al.*[4]), ST001237 (human sera, Orbitrap QE), and ST001667(mouse liver, Q-TOF data). Access of all data is described in "**Data Availability**".

LC-MS metabolomics experiments. The human plasma samples used in this study included a pooled deidentified QC sample in a vaccination cohort, NIST SRM 1950 (https://www-s.nist.gov/srmors/view_detail.cfm?srm=1950), and a commercial reference sample Qstd (Sterile Filtered Human Plasma (K2) EDTA, Equitech Bio, Inc. KERRVILLE, TEXAS). The BM21 experiment included a serial mixture of human plasma (Qstd) and vegetable juice, at the ratio of 1024:1, 256:1, 64:1, 16:1, 4:1, 1:1, 1:4, 1:16, 1:64, 1:256 and 1:1024. Along with the 11 serial mixture samples, 100% vegetable juice and 100% plasma were also included. All samples were analyzed in triplicates, while one replicate was used for data analysis in this study for simplicity. The dry extracts of unlabeled and ^{13}C labeled *E. coli* (Cambridge Isotope Laboratories, Inc.; Catalog number: MSK-CRED-DD-KIT) were reconstituted in 100 µL of ACN/H_2O (1:1, v/v) then sonicated (10 mins) and centrifuged (10 mins at 18,041 x g and 4°C) before overnight incubation at 4°C. The supernatant for each $^{12}C/^{13}C$ *E. coli* extract was collected and then prepared for LC-MS analysis. These samples were run in triplicates.
Metabolites extraction was carried out by protein precipitation technique using extraction solvent, acetonitrile:methanol (8:1, v/v) containing 0.1% formic acid and isotope labelled Trimethyl-13C3]-caffeine, [13C5]-L-glutamic acid, [15N2]-Uracil, [15N,13C5]-L-methionine, [13C6]-D-glucose and [15N]-L-tyrosine as spike-in controls. 30 µL of plasma sample was taken and 60 µL of extraction solvent was added. Extraction blanks were also prepared to remove features of non-biological origins. All samples were vortexed and incubated with shaking at 1000 rpm for 10 min at 4°C followed by centrifugation at 4°C for 15 min at 20,817 x g. The supernatant was transferred into mass spec vials and 2 µL injected into UHPLC-MS.
All samples were maintained at 4 °C in the autosampler, and analyzed using a Thermo Scientific Orbitrap ID-X Tribid Mass Spectrometer coupled to a Thermo Scientific Transcen LX-2 Duo UHPLC system, with a HESI ionization source, using positive and negative ionizations. The MS settings are: spray voltage, 3500 V; sheath gas, 45 Arb; auxiliary gas, 20 Arb; sweep gas, 1 Arb; ion transfer tube temperature, 325 °C; vaporizer temperature, 325 °C; mass range, 80-1000 Da; maximum injection time, 100 ms. The resolution was set at 120,000 in the HZV029 experiment, 60,000 in the BM21 and SZ22 experiments.

Data were acquired using hydrophilic interaction liquid chromatography (HILIC) positive and reversed phase (RP) negative polarities in full scan mode with mass resolution of 120,000

simultaneously. An AccucoreTM-150-Amide HILIC column (2.6 µm, 2.1 mm x 50 mm) and a Hypersil GOLDTM RP column (3 µm, 2.1 mm x 50 mm) maintained at 45 ºC were used for chromatographic separation. 0.1% formic acid in water and 0.1% formic acid in acetonitrile were used as mobile phase A and B respectively for RP acquisition. 10 mM ammonium acetate in acetonitrile:water (95:5, v/v) with 0.1% acetic acid as mobile phase A and 10 mM ammonium acetate in acetonitrile:water (50:50, v/v) with 0.1% acetic acid as mobile phase B were used for HILIC method. For HILIC acquisition, following gradient was applied at a flow rate of 0.55 ml/min: 0-0.1 min: 0% B, 0.10-5.0 min: 98% B, 5.00-5.50 min: 0% B and 4.5 min for cleaning and equilibration of column. For RP column, following gradient was applied at a flow rate of 0.4 ml/min: 0-0.1 min: 0% B, 0.10-1.9 min: 60% B, 1.9-5.0 min: 98% B, 5.00-5.10 min: 0% B and 4.9 min cleaning and column equilibration. The chromatographic run time was 5 min followed by 5 min washing step after each sample.

LC-MS metabolomics data processing.

Asari has default parameters with mass accuracy of 5 ppm, minimum peak height 1E5. No parameter was modified unless described specifically. The results in this paper were based on version 1.10.6.

MZmine 2.53 (or version 3.3.0) processing was performed in the following order:

1. Mass detection, using Centroid mass detector.
2. ADAP Chromatogram builder, using min group size of 6 scans, Group intensity threshold 1E3, Min highest intensity 1E4, m/z tolerance 0.001 or 5 ppm.
3. Feature detection, Chromatogram deconvolution, m/z center calculation: median, using either

   a) Wavelets (ADAP) algorithm, S/N threshold 7, S/N estimator intensity window SN, min feature height 1E4, coefficient/area threshold 100, peak duration range 0.02-1, RT wavelet range 0.02-0.5. Or

   b) Local minimum search algorithm, with Chromatographic threshold 80%, Search minimum in RT range 0.02 min, Minimum relative height 5%, Minimum absolute height 1E4, Min ratio of peak top/edge 3, Peak duration range 0.02-0.5 min.
4. Alignment, Joint Aligner, using m/z tolerance 0.001 or 5 ppm, Weight for m/z 80, Retention time tolerance 0.2 min, Weight for RT 30.

XCMS (version 3.18.0) processing was performed using the following key parameters:

CentWaveParam(peakwidth = c(1, 30), ppm = 5, noise = 1000, prefilter = c(3, 1000))

MergeNeighboringPeaksParam(expandRt = 5, ppm = 1, minProp = 0.5)

groupChromPeaks: (minFraction = 0.1, bw = 3, binSize=0.001)

The R script used in the processing is posted in our asari GitHub repository under test/.

MS-DIAL (version 4.90) processing used the following parameters:

Smoothing method: LinearWeightedMovingAverage, Smoothing level: 3, Minimum peak width: 6, Minimum peak height: 5000, Retention time tolerance: 0.05, MS1 tolerance: 0.001, Retention time factor: 0.3, MS1 factor: 0.8, Gap filling by compulsion: True.

Evaluation of feature detection. The detection of a feature requires matching the m/z values within 5 ppm and retention time within 6 seconds, but does not require unique matching. The verified 402 features in **Figure 4a** were based on three samples (batch4_MT_20210729_003G, batch4_MT_20210729_003C, batch4_MT_20210729_003K). Data were processed using Thermo Scientific Compound Discoverer (v3.3), and a list of 402 manually verified features was created by visual inspection in FreeStyle (v1.8 SP2). The manually certified features in **Figure 4b** were given in the original publication (Chen *et al.* [21]), in the three unlabeled yeast samples with negative ionization. The true features in the NIST SRM 1950 sample were manually verified, and the list of 39 m/z features is given in the GitHub repository (**Data Availability**). For the credentialed E. coli data (SZ22), the two feature tables were annotated separately using khipu (Li and Zheng[27]) to identify isotopic patterns. A pattern is considered valid when at least one M0 ion and one ion with more than one 13C labels are present in the compound group. The valid groups were combined from XCMS and asari, and [M+H]+ ions were compiled into the 643 "certified" features.

Evaluation of computational performance. Computational performance was evaluated on a desktop computer with Intel i7-8809G CPU and 32 GB of memory, running Mint Linux 20.2. The asari version was 1.10.6. The XCMS version was 3.18.0. The R script for XCMS is provided in asari repository (https://github.com/shuzhao-li/asari) under doc/ directory. The time and memory use was measured by `/usr/bin/time –v`, and "User time" was used as CPU time (equivalent to CPU time used on a single core).

Data structures in asari. Examples of key data structures are given here as Python dictionaries (JSON compatible):

```
sample: {
    'input_file': '',
    'list_scan_numbers': [],
    'list_retention_time': [],
```

```
    ‘rt_cal_dict’: {},
    ‘list_mass_tracks’: [],
    }

mass_track: {
    'id_number': 999,
    'mz': 230.01808166503906,
    'intensity': array([
        19265,  23414,  19809,  24195,  27025,  32111,  37948,  17387,
        33759,  32037,  24933,  15248,  30890,  24147,  37816,  52205,
        19562,  44433,  40049,  40032,  66720,  37805,  41621,  51155,
        52528,  41309,  51672,  36679,  63472,  55880,  63660,  68534,
        61723,  68821,  39000,      0,      0,      0,      0,      0,
            0,  59430,  91510, 220216, 254759, 273220,  64931,  25042,
            0,      0,  18063,  16858,  37267,  34924,  35216,  44651,
        36903,  51591,  31158,  31455,      0,      0,  58454,  48568,
        47294,  37701,  59255,  45277,  32742,  57048,  59220,  55310,
        71600,  51937,  52947,  54520,  46489,  56674,  62587,  55151,
        61771,  76765,  7699, ...,
            0,      0,      0,      0,      0,      0,      0,  10539,
            0,      0,      0,      0,      0,      0,   8404,      0,
            0,      0,      0,      0,      0,      0,      0,      0,
            0,   8164,      0,      0,      0,      0,      0])
}

feature:{
        "id_number": "F3943",
        "parent_masstrack_id": 2791,
        "mz": 313.23848724365234,
        "apex": 156,
        "left_base": 147,
        "right_base": 168,
        "rtime": 150.344,
        "rtime_left_base": 145.302,
        "rtime_right_base": 157.107,
        "peak_area": 153629884,
        "height": 18384026,
        "representative_intensity": 153629884,
        "snr": 217,
        "goodness_fitting": 0.9769980444055228,
        "cSelectivity": 1.0,
    },

empirical_compound: {
        "interim_id": "kp480_314.2458",
        "neutral_formula_mass": 314.245709575,
        "neutral_formula": "C18H34O4",
        "Database_referred": [],
```

```
"identity": [],
"MS1_pseudo_Spectra": [
{
    "apex": 156,
    "peak_area": 153629884,
    "height": 18384026,
    "left_base": 147,
    "right_base": 168,
    "goodness_fitting": 0.9769980444055228,
    "cSelectivity": 1.0,
    "parent_masstrack_id": 2791,
    "mz": 313.23848724365234,
    "snr": 217,
    "id_number": "F3943",
    "rtime": 150.344,
    "rtime_left_base": 145.302,
    "rtime_right_base": 157.107,
    "representative_intensity": 153629884,
    "id": "F3943",
    "isotope": "M0",
    "modification": "M-H-",
    "ion_relation": "M0,M-H-",
    "parent_epd_id": "kp480_314.2458"
},
{
    "apex": 156,
    "peak_area": 26869245,
    "height": 3362120,
    "left_base": 150,
    "right_base": 168,
    "goodness_fitting": 0.9598939375019998,
    "cSelectivity": 1.0,
    "parent_masstrack_id": 2805,
    "mz": 314.2418899536133,
    "snr": 332,
    "id_number": "F4045",
    "rtime": 150.344,
    "rtime_left_base": 146.982,
    "rtime_right_base": 157.107,
    "representative_intensity": 26869245,
    "id": "F4045",
    "isotope": "13C/12C",
    "modification": "M-H-",
    "ion_relation": "13C/12C,M-H-",
    "parent_epd_id": "kp480_314.2458"
},
{
    "apex": 161,
```

```
            "peak_area": 10973710,
            "height": 1116800,
            "left_base": 155,
            "right_base": 173,
            "goodness_fitting": 0.588704815611131,
            "cSelectivity": 1.0,
            "parent_masstrack_id": 2616,
            "mz": 295.2277374267578,
            "snr": 46,
            "id_number": "F4216",
            "rtime": 153.157,
            "rtime_left_base": 149.783,
            "rtime_right_base": 159.93,
            "representative_intensity": 10973710,
            "id": "F4216",
            "ion_relation": "M-H2O-H[-]"
        }
        ],
        "MS2_Spectra": [],
        "list_matches": [
        [
            "C18H34O4_314.24571",
            "M-H[-]",
            2
        ],
        [
            "C18H36O5_332.256274",
            "M-H2O-H[-]",
            1
        ]
        ]
    }
```

Step-by-step description of asari default processing workflow.

1. Build sample registry;
   see `workflow.process_project`, `workflow.register_samples`.

2. Mass track construction for each sample;
   see `chromatograms.extract_massTracks_`.

   a. Get all MS1 spectra from a data file, as a list of [(m/z, scan number, intensity), ...]. Index to a dictionary by int(mz * 1000) for efficient retrieval. This is an mzTree.

   b. Create a list of data bins from mzTree. Each bin starts with a value in the mzTree, which has a m/z range around 0.001 and is filtered by minimal required

scan number. If two bins are adjacent by 0.001 or within tolerance ppm, they are merged. See `chromatograms.get_thousandth_bins`.

c. Build mass tracks per data bin. If the m/z range in a data bin is within 2 x tolerance ppm, the bin leads to a single mass track.

Else, a nearest neighbor (NN) clustering is performed to establish the number of mass tracks. The NN clustering assigns each data point to its nearest "peak mz value". The "peak mz values" are determined by finding peaks in the m/z value distribution, with the requirement that the two peaks need to be separated by the mz tolerance minimally. The m/z value distribution is approximated by a histogram for computing efficiency.
See `mass_functions.nn_cluster_by_mz_seeds`.
A mass track is defined by a consensus m/z value and a list of intensity values. The consensus m/z value is taken as the mean of (median m/z and the m/z at highest intensity). This avoids instable values caused by outliers. When multiple data points exist in the same scan (same RT), max intensity is used. This cleans up duplicate mass peaks from the centroiding process. The mass track is of full RT range, with zeros inserted where intensity is missing.
See `chromatograms.extract_single_track_fullrt_length`.

d. Establish anchor mass tracks by finding m/z differences that match to either 13C/12C isotopes or Na/H adducts. These anchors are considered of higher confidence and prioritized in m/z alignment.

e. For all samples, use parallel processing;
See `workflow.batch_EIC_from_samples_`.

3. Alignment of mass tracks across samples, resulting in the MassGrid;
   See `CompositeMap.construct_mass_grid`.

   a. The sample with the highest number of anchor mass tracks is designated as the reference sample, unless a user specifies a reference sample via input parameters. See `ext_Experiment.get_reference_sample_id`.

   b. If the sample number is no more than a predefined parameter ('project_sample_number_small', default 10), this is considered a small study and a pairwise alignment is performed. When a new sample is aligned to the reference list of m/z values (initially from the reference sample), the anchor mass tracks are compared and mapped first. If there is a systematic difference greater than a preset value (default 1 ppm), all m/z values in the sample are recalibrated against the reference. Because the anchors are of higher confidence and mostly have well resolved m/z values, their alignment has less chance for errors. By completing the alignment of anchors first, the remaining m/z values (significantly fewer than the total features) do not compete against the anchors during their

alignment, leading to cleaner results.
See `MassGrid.build_grid_sample_wise`, `MassGrid.add_sample`.

c. Else, for a larger study, the mass alignment is performed by the same NN clustering method that is used in initial mass track construction.
See `MassGrid.build_grid_by_centroiding`, `MassGrid.bin_track_mzs`

4. Retention time alignment

   a. The reference sample was established prior to mass alignment. A set of landmark elution peaks are determined in this reference sample by the criteria: mSelectivity > 0.99, min_peak_height is satisfied (default 1e5), prominence > 20% of peak height and the peak is the only peak on its mass track. These are our "selected_reference_landmark_peaks".
   See `set_RT_reference`, `peaks.quick_detect_unique_elution_peak`.

   b. For each of the remaining samples, a set of good peaks are selected using the same criteria as above, but limited to from the mass tracks already aligned to the selected_reference_landmark_peaks. They constitute the "good_landmark_peaks" specific to a sample.
   See `CompositeMap.calibrate_sample_RT`.

   c. Perform a LOWESS (Locally Weighted Scatterplot Smoothing) regression to obtain a function to describe the relationship of the RT values between good_landmark_peaks and selected_reference_landmark_peaks. To prevent spurious results from the regression, we add 10% extension out of both ends as boundaries that the regression must converge to. In practice, the function extrapolates to a dictionary to map all scan numbers between the current sample and the reference sample. The dictionary skips numbers that are identical between the two samples to save memory and computing. The resulting "rt_cal_dict" is a correspondence between sample_rt_numbers and reference_rt_numbers. See `chromatograms.rt_lowess_calibration`

   d. Loop through all remaining samples to obtain the RT calibration function (rt_cal_dict) per sample.

5. Building the composite map

   a. Now that we have established both the m/z alignment in MassGrid and RT alignment between samples, they are linked in the `CompositeMap` class.

   b. Each LC-MS feature should be on a specific location on the composite map (with a consensus m/z and RT). A feature can be conceptualized as a pattern that is repeated in multiple samples. Thus, the pattern will persist and be even enhanced when the signals from multiple samples are superimposed. This

concept is implemented into "composite mass tracks", where the intensity values are summed on corresponding mass tracks across all samples after RT calibration. The mass tracks are intensity vectors of the same length, based on scan numbers, after retention time alignment.
See `CompositeMap.build_composite_tracks`.

6. Detection of elution peaks (features)

   Instead of detecting the same peaks in every sample, asari detects an elution peak only once on the composite mass tracks. Because a composite mass track represents all samples, an elution peak here is equivalent to a feature in the experiment. The shape and prominence of a peak matter in relation to its surroundings. Some instruments generate higher intensity numbers than others arbitrarily. The absolute intensity number is not the most important; a peak is distinctive as long as its shape is good and its height is well above the noise level. We may state that all good peaks look similar. It is the "scaling" factor that differs between metabolites and between platforms. The peak detection in asari is largely dependent on the noise levels. The default parameters are functional over a large range and updated based on statistical analysis of the mass track.
   See `peaks.audit_mass_track`, `peaks.stats_detect_elution_peaks`.

   a. If the max intensity of a mass track is higher that a preset ceiling (1E8), the mass track is rescaled under the preset ceiling for the purpose of peak detection. This serves a normalization purpose to make peak detection parameters robust. Because the composite mass tracks sum up the cumulative intensity of all samples, the intensity could be exceedingly high in large studies. But the detection of elution peaks on composite mass tracks only needs to determine the apex and peak boundaries. The useful quantitative value is the peak area reported on individual samples, not on a composite mass track. After peak detection, the peak height is scaled back using the same scaling factor.

   b. If the median intensity on a mass track is below the preset min_intensity_threshold (default 1e3 for Orbitrap data), this is a low-intensity track. Both baseline level and noise level are set to min_intensity_threshold.

   c. Else, if over half the data points are above min_intensity_threshold and the median intensity is higher than 10 times of preset min_peak_height (default 1e5 for Orbitrap data), detrend (scipy.signal.detrend) is performed on the mass track. Detrend is a regression method to ensure the baseline is not significantly rising or decreasing over the chromatography. It is a computationally expensive operation and only required for high-signal and high-noise data.

   d. If a track is not low-intensity (see b), the bottom signals are taken as intensity values below the lower quartile plus min_intensity_threshold. The constant of min_intensity_threshold makes this method stable, even when zeros dominate

the track. Here, the baseline level and noise level are assigned as the mean and standard deviation of the bottom signals, respectively.

e. Smoothing (chromatograms.smooth_moving_average) is applied when the noise level is higher than 1% of max intensity and max intensity is lower than 10 times of the preset min_peak_height. For low noise or high intensity tracks, smoothing is not needed.

f. The mass track is subtracted by a filter (i.e. baseline + noise level). This creates multiple segments of positive intensity values, because some data points are below the filter. Peak detection is performed on the separate regions, because i) it is faster to skip very low signals, ii) this reduces the chance of dealing with multiple peaks simultaneous, and iii) this allows further determination of proper peak prominence based on local signal levels.

g. Peak detection on a segment of signals. Asari uses a simple local maxima method (scipy.signal.find_peaks), with prominence control that is dynamically determined on each mass track then each segment. Prominence is the vertical distance from a peak top to its lowest contour line. Prominence is calculated in a series of steps: the initial value is 1/3 of min_peak_height; then the greater of prominence and the noise level of a track is used as the new prominence; if the segment is high-intensity and high-noise, the greater of prominence and 5% of max intensity is used as the new prominence. Another parameter, min_peak_height, applies to this method. The parameter min_timepoints is used for controlling both distance between peaks and peak width. The prominence is computed on a sliding window size (default 25 scans). The parameters other than prominence are fairly robust at their default values.
See `peaks.stats_detect_elution_peaks`, `peaks.detect_evaluate_peaks_on_roi`.

h. The detected elution peaks are evaluated for peakshape, cSelectivity and SNR. The default filters are set low for these values, so that users can decide on their filtering on the feature table.
See `peaks.evaluate_gaussian_peak_on_intensity_list`, `peaks.__peaks_cSelectivity_stats_`, `peaks.compute_noise_by_flanks`. The peaks passing the thresholds (default SNR > 2 and peakshape > 0.5) are reported in a JSON format, with link to the composite mass track identifier.

7. Feature assignment and annotation

a. The elution peaks detected from composite mass tracks are stored in CompositeMap.FeatureList (they are experiment-wide features). The retention time is converted from scan numbers to seconds (see `CompositeMap.global_peak_detection`). The features have peak positions and boundaries on the composite mass tracks, which are translated to positions

on the individual samples, via the RT alignment dictionaries (see `CompositeMap.extract_features_per_sample`). The sample specific peak areas (default method as the sum of all intensity values within peak boundaries) are recorded into CompositeMap.FeatureTable.

b. The pre-annotation is done via another package khipu (https://github.com/shuzhao-li-lab/khipu), where isotopes and adducts are grouped into empirical compounds.

c. The empirical compounds are searched against a known compound database (default HMDB 4) via another package JMS (https://github.com/shuzhao-li/JMS). The matched isomers (not distinguished without additional information) and formulas are included in asari output.
See `experiment.ext_Experiment.annotate`.

8. Data export

The output directory by asari bears a time stamp, not to overwrite existing data. The recommended feature table is `preferred_Feature_table.tsv`. All peaks are kept in `export/full_Feature_table.tsv`. Annotation is exported into both JSON and tsv formats. See `experiment.ext_Experiment.export_peak_annotation`. MassGrid is exported as a csv file. The composite map is exported as a pickle file, which is used by the visual dashboard.

Supplementary Tables

Supplementary Table 1. Pairwise, unambiguously matched features between processing tools on the HZV029 dataset. MZmine is using the ADAP wavelets algorithm for elution peak detection, and MZmine(L) is based on the local minimum search algorithm. Processing parameters and software versions are given in the Methods section. In the comparison between the two result tables, a feature is considered matched when the m/z difference is within 5 ppm and retention time is within 6 seconds; if multiple matches are found within the parameters, the pair with the closest retention time is chosen. Alternative methods of choosing among multiple matches by closest m/z values produced similar results.

HZV029q (184 files of repeated QC sample, positive ionization)

XCMS	*MZmine*	*MZmine(L)*	*MS-DIAL*	*asari*	
10901	6186	6227	5421	5360	*XCMS*
	42099	17223	14286	13164	*MZmine*
		24837	12527	11963	*Mzmine(L)*
			54863	10624	*MS-DIAL*
				22440	*asari*

Supplementary Table 2. Pairwise, unambiguously matched features between processing tools on the Yeast2021 dataset. Methods are the same as in Supplementary Table 1.

Yeast2021 (3 files, yeast culture extracts, negative ionization)

XCMS	*MZmine*	*Mzmine(L)*	*MS-DIAL*	*asari*	
6043	4728	4364	2609	3013	*XCMS*
	11290	6498	2939	3435	*MZmine*
		18153	2760	3395	*Mzmine(L)*
			4166	3108	*MS-DIAL*
				5341	*asari*

Supplementary Figures

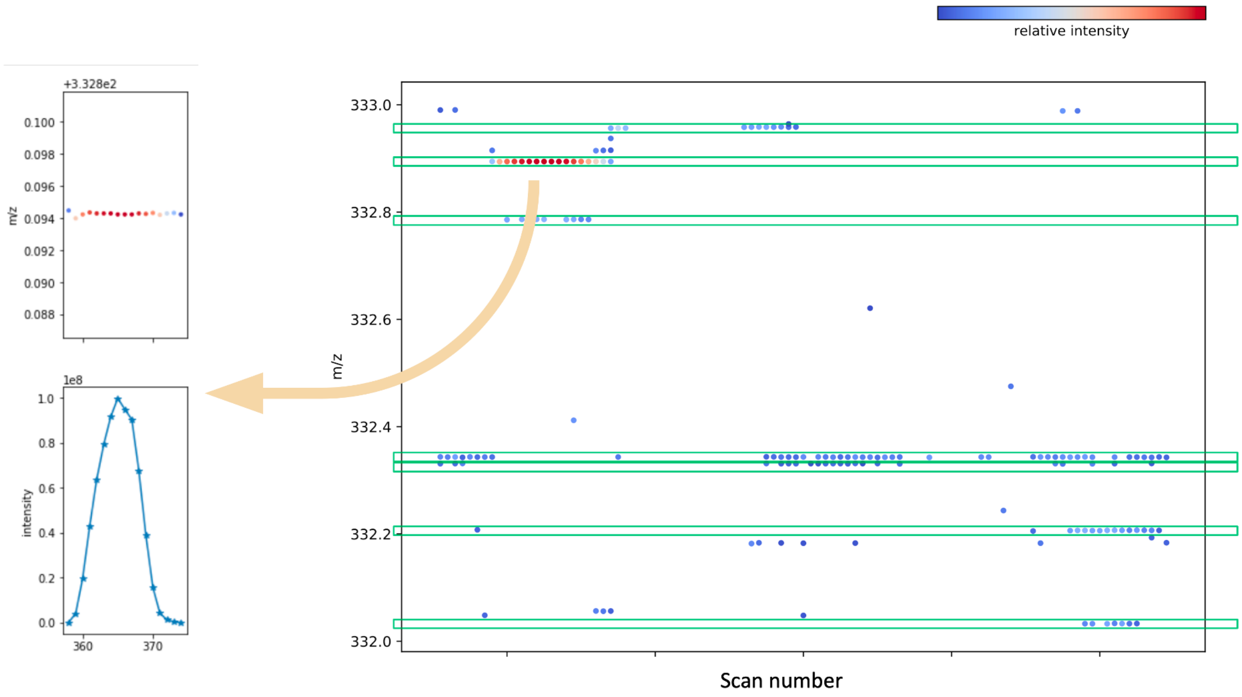

Supplementary Figure 1. Illustration of mass tracks in a single sample. The region has seven mass tracks marked by green boxes spanning horizontally, each of a unique m/z value. A peak is detected from the track indicated by the yellow arrow.

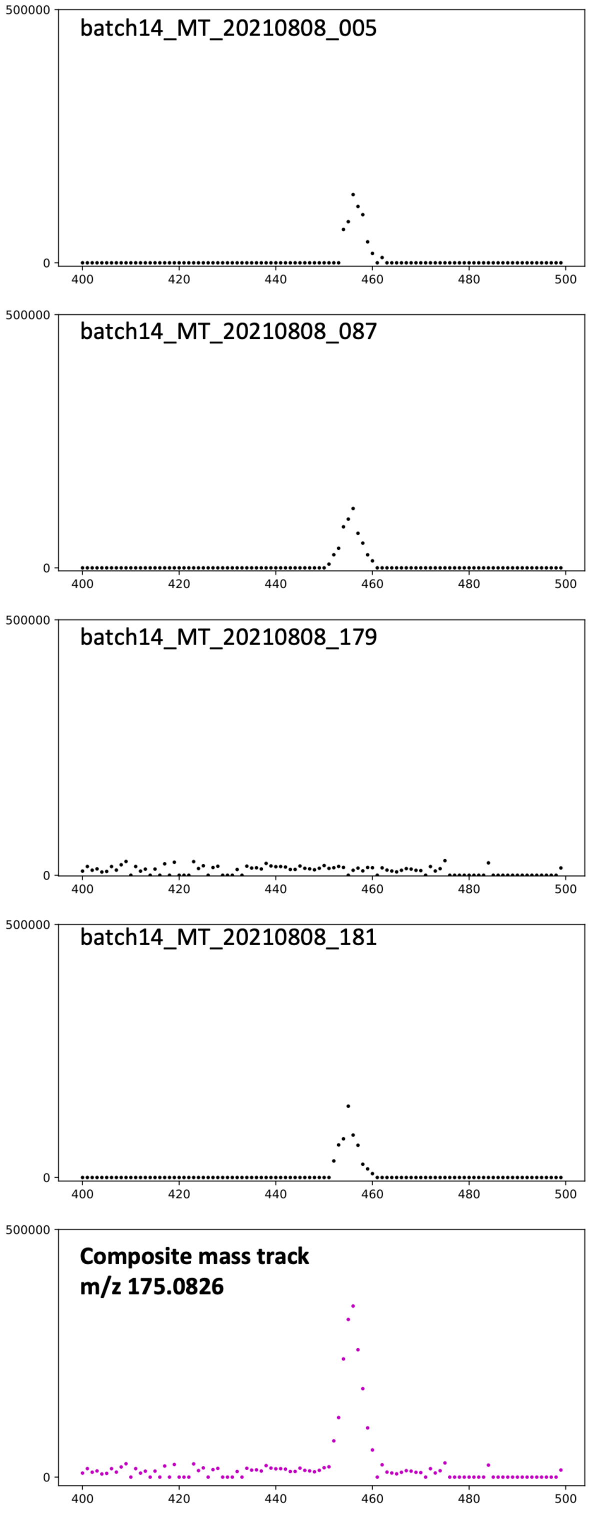

Supplementary Figure 2. Composite mass track enhances peak detection by aggregating signals from all samples. An example is shown at m/z 175.0826 in the MT02 dataset. Four individual samples are shown on top, and the composite mass track at bottom.

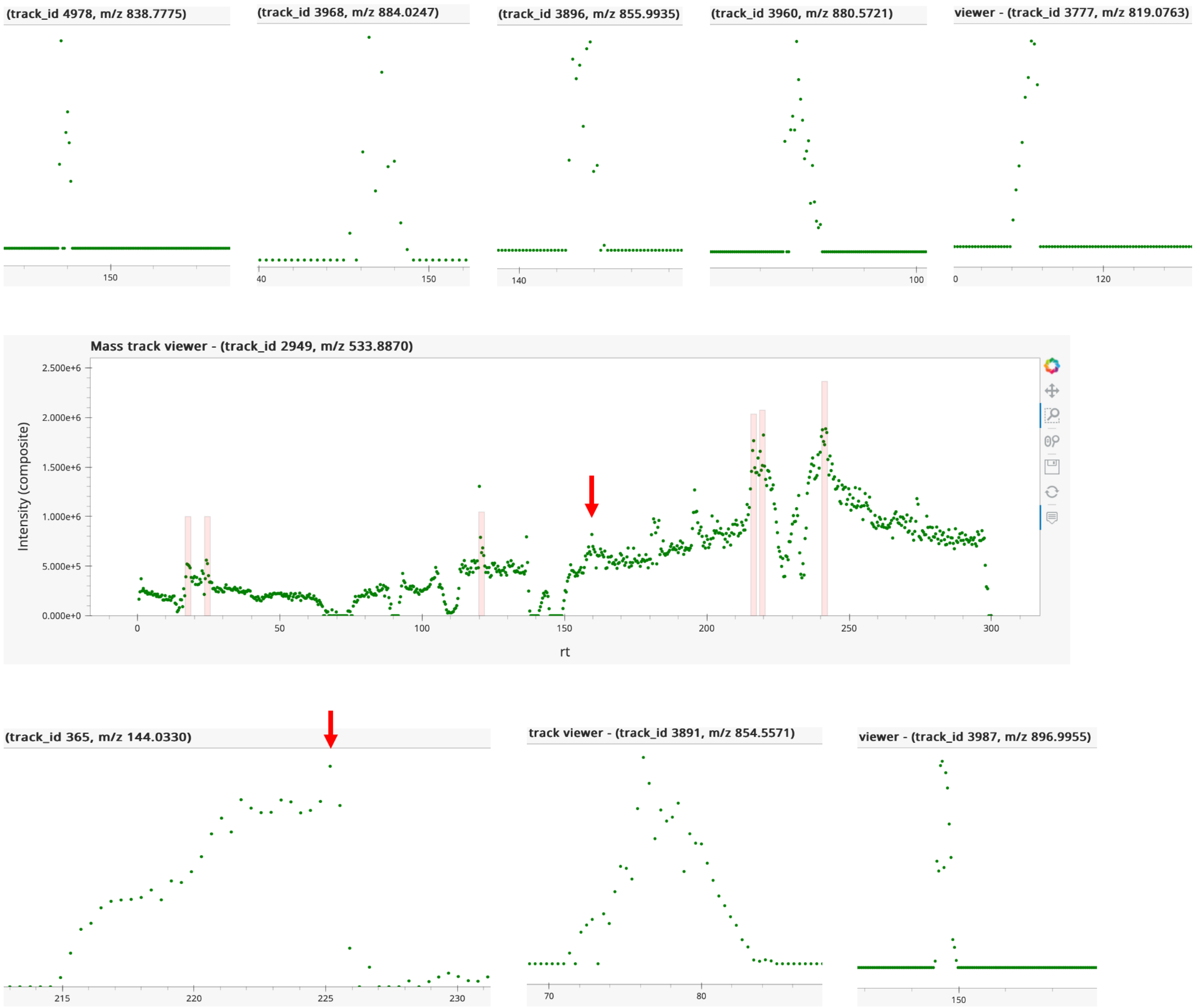

Supplementary Figure 3. Manually verified features that are not reported by asari in the HZV029 dataset (corresponding to Figure 4a). Part or full mass track is shown. The red arrow indicates the missed features in complex tracks.

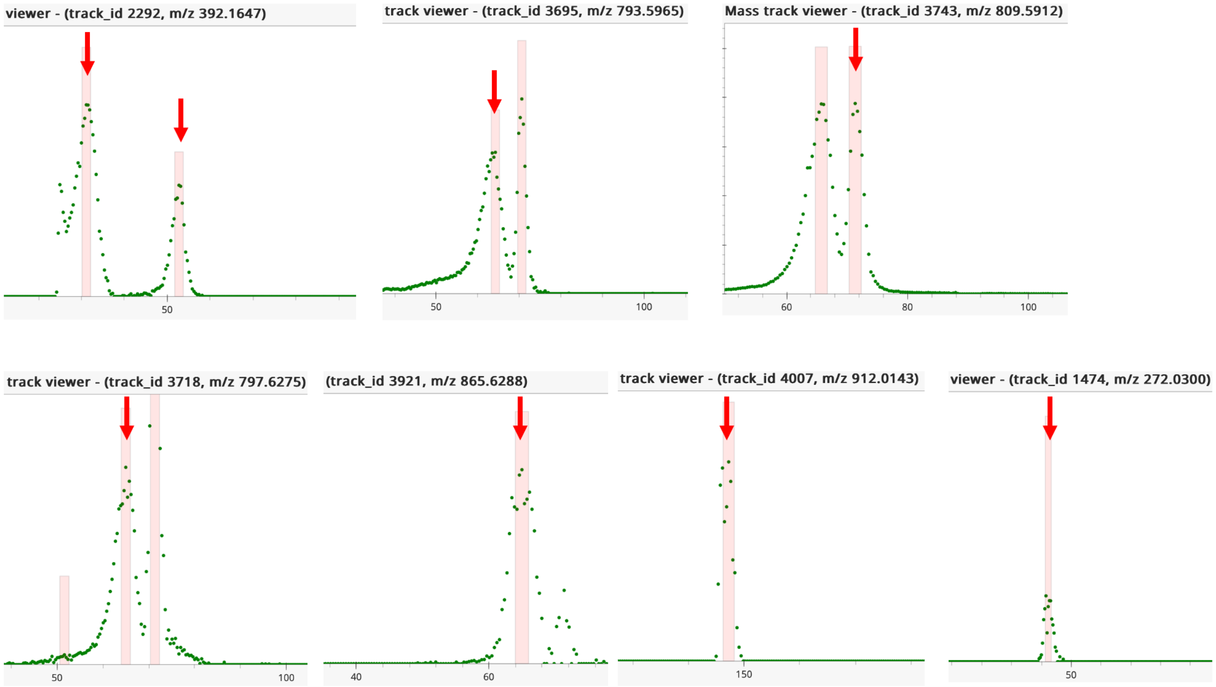

Supplementary Figure 4. Manually verified features that are not reported by MS-DIAL in the HZV029 dataset (corresponding to Figure 4a). Partial mass tracks are shown. The red arrows indicate the missed features. These features were all detected by asari.

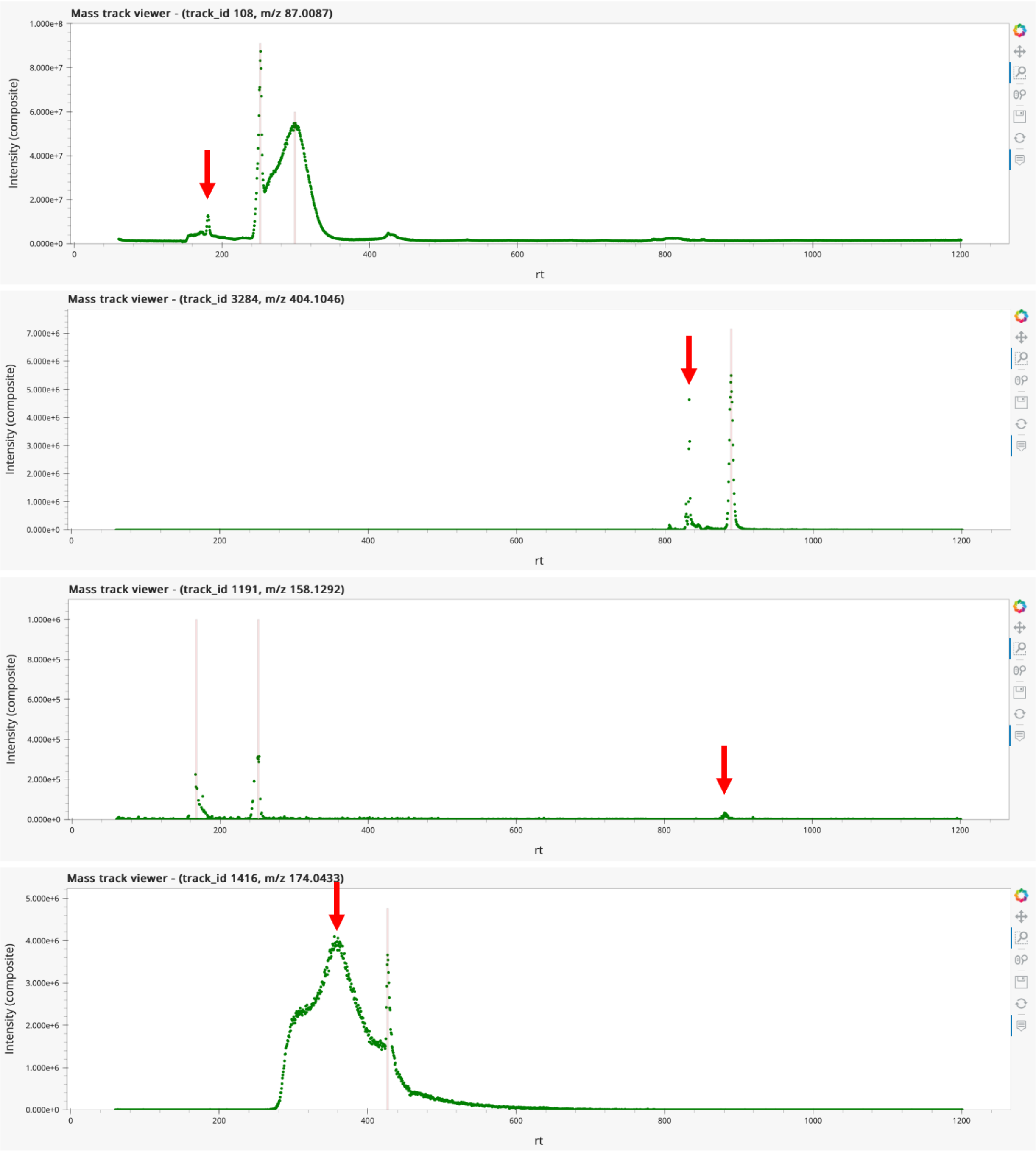

Supplementary Figure 5. Four manually verified features are not reported by asari in the Yeast2021 dataset (corresponding to Figure 4b). The red arrows indicate the missed features. The top two were due to too few valid data points in a peak. The third case was caused by peak height below threshold and lowering the threshold enabled detection of the peak. The bottom case was due to high local noise.

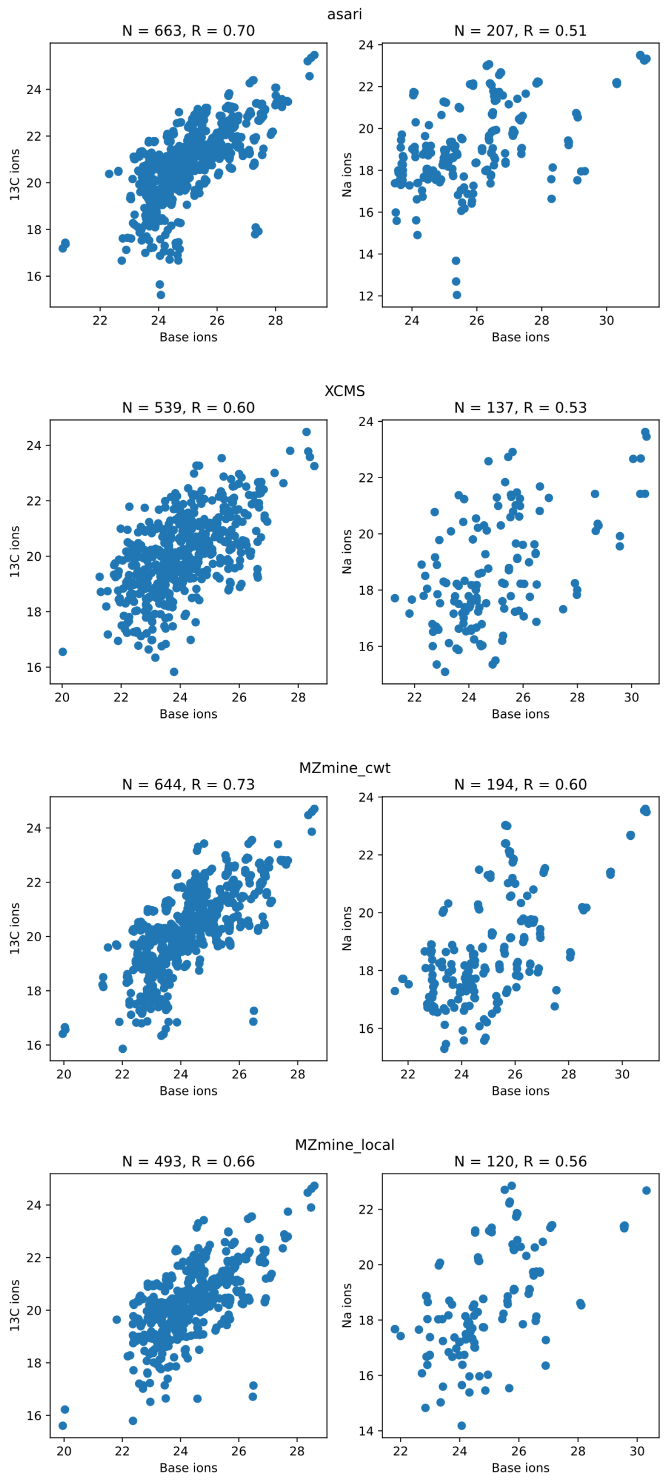

Supplementary Figure 6. Correlation of 13C isotopologues and Na+ adducts to the known compounds in the Yeast2021 dataset (corresponding to Figure 4b). The 13C and Na+ adduct peaks are retrieved by the m/z distance of 1.003355 and 21.9820, respectively, and the smallest difference in retention time under 2 seconds. The "N" indicates the number of nonzero peaks found in all three samples, "R" Pearson correlation coefficient, and intensity values at log2 scale. Source data are provided as a Source Data file.

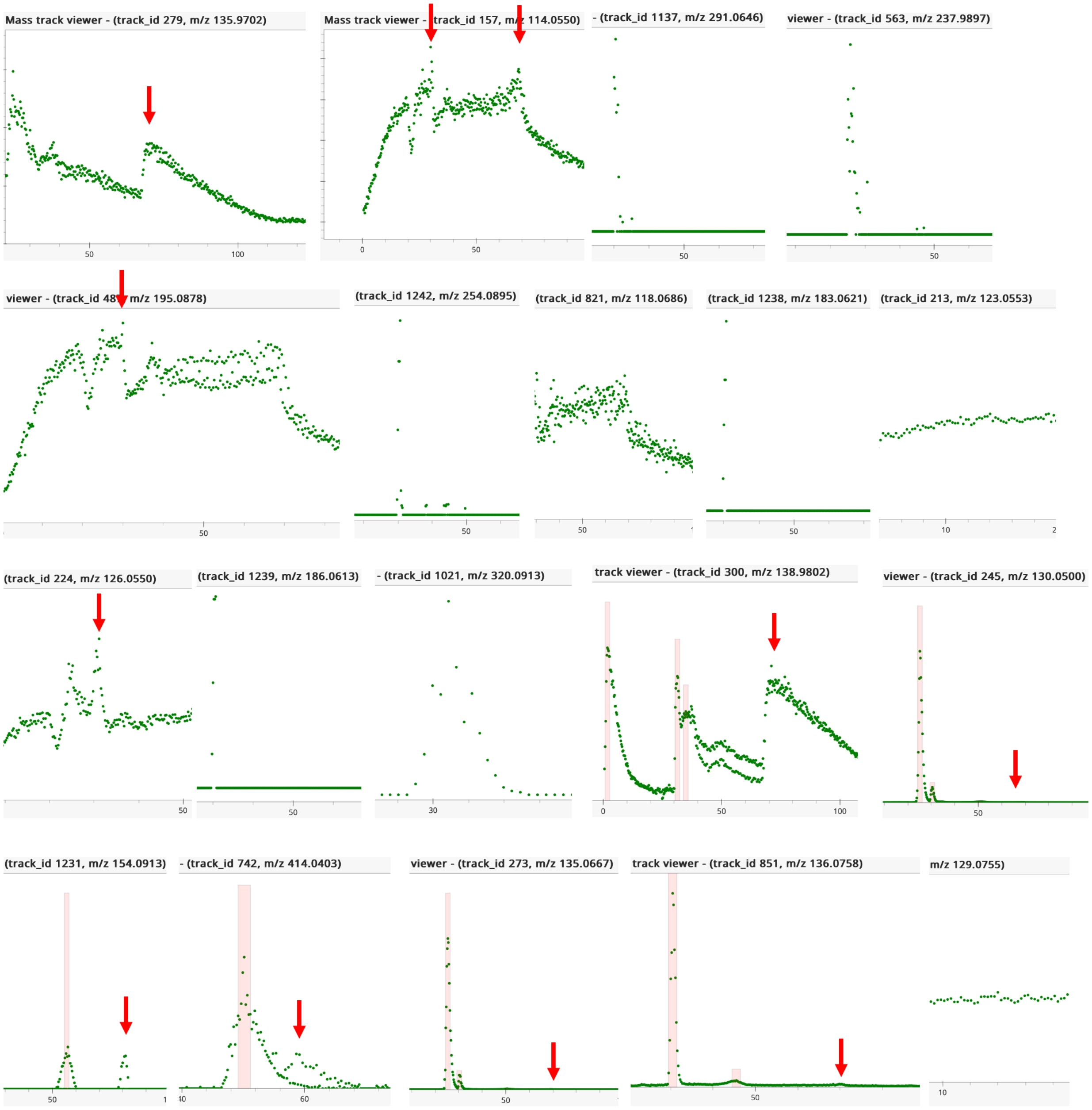

Supplementary Figure 7. Features not reported by asari in the SZ22 E. coli dataset (corresponding to Figure 4c). Partial mass tracks are shown. The red arrow indicates the missed features.

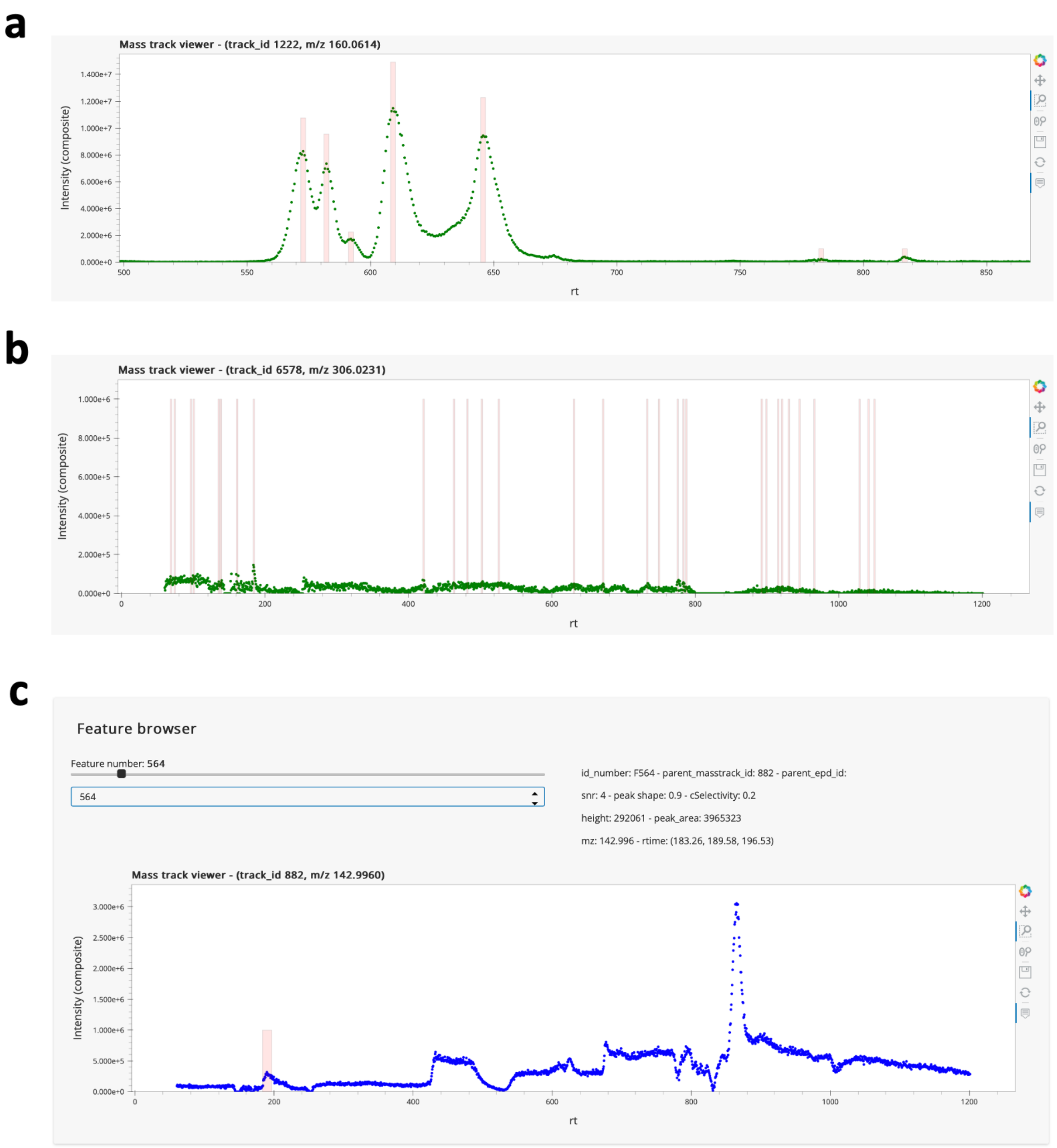

Supplementary Figure 8. Example chromatograms for illustrating issues in peak detection.

a) Features detected by asari are marked by pink vertical line. Corresponding to the top right panel in Figure 5A. **b**) Inappropriate parameters can lead to many low-quality peaks. Each pink vertical line indicates a peak. **c**) The chromatography shows higher baseline in later elution time. This last image is from Feature Browser in the asari dashboard, which shows only the current, not all, features on the mass track.

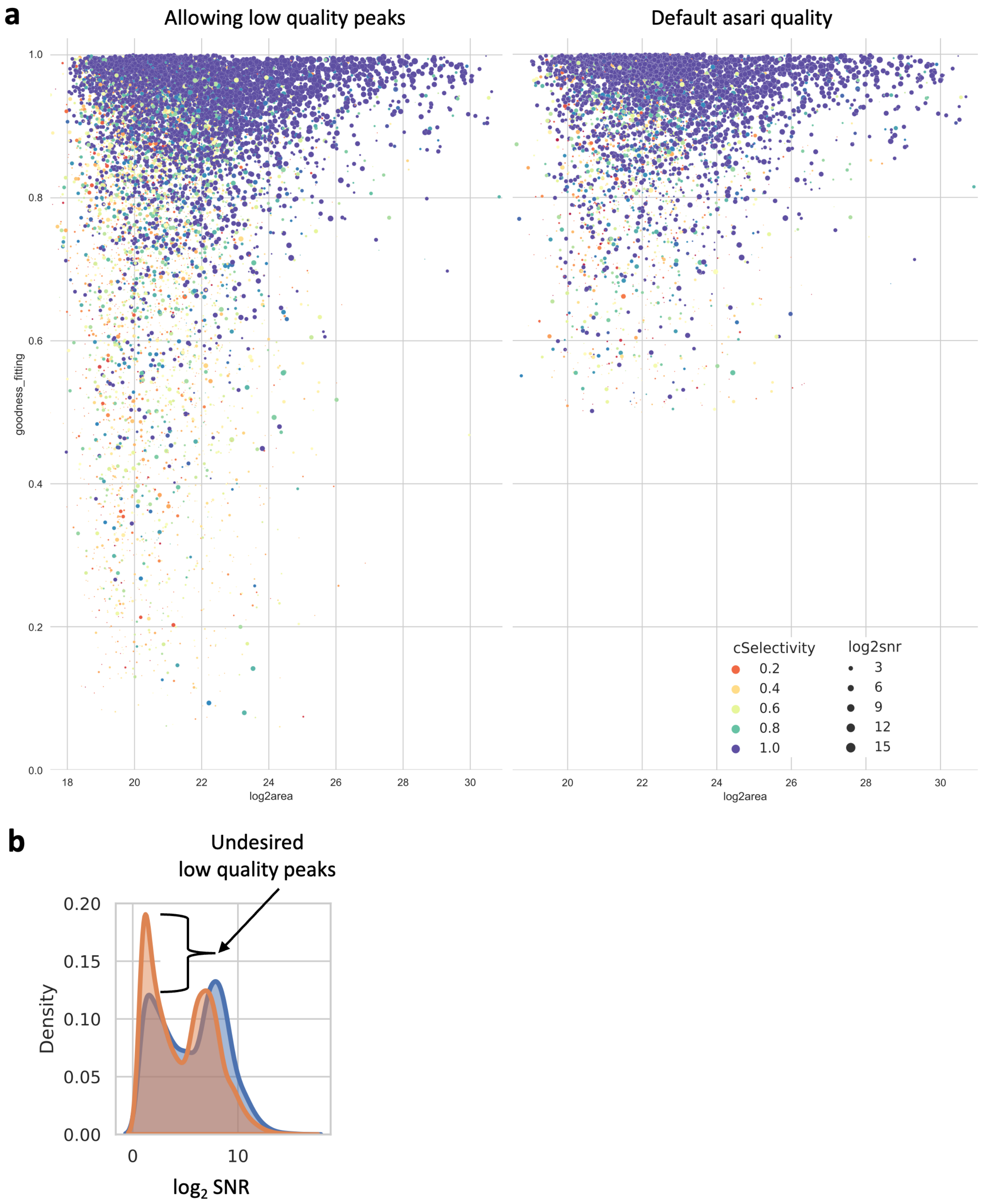

a
Allowing low quality peaks
Default asari quality
goodness_fitting
log2area
cSelectivity
0.2
0.4
0.6
0.8
1.0
log2snr
3
6
9
12
15
b
Undesired
low quality peaks
Density
log2 SNR

Supplementary Figure 9. Example of QC overview with many low-quality peaks.

a) Features extracted by asari using very low QC values (min peak height 5E4, SNR > 1.2, peakshape > 0.05) on the left to simulate a dataset with many undesired features (total 10306 features), in contrast with features from default parameters on the right (same as Figure 6C but using different color scheme and rounding precision).

b) The undesired features show at the lower range of SNR in kernel density plot. Data from default parameters are in blue and lower parameters in pink. Source data are provided as a Source Data file.

Supplementary References:

1. Li, S.; Zheng, S., Generalized Tree Structure to Annotate Untargeted Metabolomics and Stable Isotope Tracing Data. *Anal Chem* **2023,** *95* (15), 6212-6217.
2. Wishart, D. S., Metabolomic data exploration and analysis with the human metabolome database. In *Computational Methods and Data Analysis for Metabolomics*, Springer: 2020; pp 165-184.
3. Chen, L.; Lu, W.; Wang, L.; Xing, X.; Chen, Z.; Teng, X.; Zeng, X.; Muscarella, A. D.; Shen, Y.; Cowan, A.; McReynolds, M. R.; Kennedy, B. J.; Lato, A. M.; Campagna, S. R.; Singh, M.; Rabinowitz, J. D., Metabolite discovery through global annotation of untargeted metabolomics data. *Nat Methods* **2021,** *18* (11), 1377-1385.
4. Delabriere, A.; Warmer, P.; Brennsteiner, V.; Zamboni, N., SLAW: A Scalable and Self-Optimizing Processing Workflow for Untargeted LC-MS. *Anal Chem* **2021,** *93* (45), 15024-15032.
